# Monitoring and Evaluating Progress towards Universal Health Coverage in Singapore

**DOI:** 10.1371/journal.pmed.1001695

**Published:** 2014-09-22

**Authors:** Kelvin Bryan Tan, Woan Shin Tan, Marcel Bilger, Calvin W. L. Ho

**Affiliations:** 1 Policy Research and Economics Office, Ministry of Health, Singapore; 2 Saw Swee Hock School of Public Health, National University of Singapore, Singapore; 3 Health Services & Outcomes Research, National Healthcare Group, Singapore; 4 Health Services & Systems Research, Duke-National University of Singapore, Singapore; 5 Centre for Biomedical Ethics, Yong Loo Lin School of Medicine, National University of Singapore, Singapore; 6 Regulatory Policy and Legislation Division, Ministry of Health, Singapore; 7 Ethox Centre, Nuffield Department of Population Health, University of Oxford, Oxford, United Kingdom

## Abstract

This paper is a country case study for the Universal Health Coverage Collection, organized by WHO. Woan Shin Tan and colleagues illustrate progress towards UHC and its monitoring and evaluation in Singapore.

*Please see later in the article for the Editors' Summary*

This paper is part of the PLOS Universal Health Coverage Collection. This is the summary of the Singapore country case study. The full paper is available as Supporting Information file Text S1.

## Background

The World Health Organization defines universal health coverage (UHC) as a situation in which all people who need health services receive them, without incurring financial hardship. UHC is currently perceived as a crucial component of sustainable development and listed as one of the possible goals of the post-2015 development agenda.

The Republic of Singapore is an island-state in southeast Asia. Promoting UHC has been an important part of Singapore's overall development strategy. Within a span of 50 years since achieving independence, investment in providing better housing, clean water, improved sanitation, and good education combined with better nutrition has enabled Singapore to improve the health status of its population [1]. Early investments in health promotion, prevention, and public education played an important role in raising the life expectancies of Singaporeans.

## Universal Health Coverage: The Policy Context

Promoting UHC has been an important part of Singapore's overall development strategy, with a strong policy focus on the promotion, prevention, and treatment of non-communicable diseases (NCDs).

The philosophy behind the Singapore health care financing system is a shared responsibility among individuals and families, insurers, and the government. Individuals and families should have a role in living healthily and in saving for future health care expenditures while health care providers are incentivized to deliver cost-effective care. In addition, insurers need to mitigate the financial risk associated with illness, and the government is responsible to provide a safety net.

To avoid the drawback of “free” medical services stimulating insatiable demand and to ensure longer-term financial sustainability of the health care system, copayments are a key feature of the Singapore health care system. A financing system comprising medical savings (Medisave), health insurance (MediShield), and government subsidies helps ensure that these copayments are affordable and do not deter people from seeking appropriate medical treatment.

## Monitoring and Evaluation for UHC

While there is no specific UHC monitoring framework, indicators on accessibility, quality, and affordability of health care for Singaporeans are regularly tracked and reported to Parliament as part of the Key Performance Indicators (KPIs) for the Ministry of Health [2]. The list of indicators includes many of the potential tracer indicators for both health-related Millennium Development Goals (e.g., vaccination coverage for diphtheria and measles) and chronic conditions and injuries (CCI) (premature mortality from cancer, ischemic heart disease, and stroke and prevalence of obesity, diabetes, psychiatric morbidity) as recommended by the World Health Organization and World Bank. The KPIs also include many “hardwired” equity considerations, including access in terms of waiting times to subsidised primary care and specialist services, and Medisave and MediShield coverage for subsidised inpatient services, which are used by the lowest 40% of income distribution.

## Progress towards UHC in Singapore

Between 1990 and 2010, there has been a rapid improvement in life expectancy, and a reduction in premature mortality from cancer, ischemic heart disease, and stroke. Singapore also rose in an international ranking of healthy life expectancy (HALE) from eleventh to second for males, and from 14 to four for females [3]. These outcomes have been achieved with spending of approximately 4% of the gross domestic product (GDP) on health expenditures.

Treatment coverage for chronic diseases (diabetes, 97%; hypertension, 97%; high blood cholesterol, 87%) is generally high and is effective at controlling the condition in approximately two-thirds of cases. While UHC may not equalise the prevalence of diseases across socioeconomic status (SES) because of the influence of various determinants of health, it should ensure that intervention coverage is equal across SES. On the basis of the results from the latest National Health Survey, service coverage and treatment outcomes for individuals with diabetes and high blood cholesterol have improved for individuals with lower educational attainment. For hypertension, service coverage was better for individuals with fewer years of education but outcomes were worse. For cancer, knowledge and utilisation of screening increases with educational attainment. (Text S1)

Affordability indicators showed that MediShield coverage increased from 80% to 92% (Text S1). In November 2013, the Ministry of Health further announced the MediShield scheme would be reviewed to ensure universal coverage. Not accounting for means-tested subsidies provided to the poorest individuals, household out-of-pocket payments relative to income amount to 4.3% to 4.5% across all quintiles (Text S1).

## Conclusions and Recommendations

The example of Singapore illustrates that even for a country with an extensive health care system, monitoring of service coverage and financial protection still remains highly important. Singapore's experience also shows that the choice of appropriate indicators will have to evolve as countries go through different phases of socioeconomic development and epidemiological change. Nevertheless, for the purpose of benchmarking and sharing across countries, it is also important to have a common set of indicators.

Moving ahead, the key challenge that Singapore faces is ensuring that good health outcomes continue to be achieved with an ageing population and projected increases in chronic conditions. To keep UHC financially sustainable, Singapore relies on income and service-differentiated patient copayments. These copayments have to be continually adjusted to make certain that they do not discourage use of important primary care and prevention that contribute in lowering the risk factors for these costly chronic conditions. The close monitoring of SES gradients in risk factors and medical treatment that we report in this paper will thus be important. If Singapore is successful in ensuring that these gradients do not deteriorate, this aspect of Singapore's health financing system could be instructive for other countries.

## Ethics Note

Ethical approval to conduct the National Health Survey was obtained from Singapore's Health Promotion Board Ethics Committee and written consent was obtained from all participants before they took part in the survey.

## Supporting Information

Text S1 **The full country case study for Singapore.**
(DOCX)

## Abbreviations

SES: socioeconomic status
UHC: universal health coverage
