# Supplementary material for: Monitoring and Evaluating Progress towards Universal Health Coverage in Singapore
Source: PLoS Med. 2014 Sep 22;11(9):e1001695. doi: 10.1371/journal.pmed.1001695 (PMC4171371; doi:10.1371/journal.pmed.1001695)
Supplement: Text S1 — The full country case study for Singapore. (DOCX) [file pmed.1001695.s001.docx]

**Full Case Study: Monitoring and Evaluating Progress towards Universal Health Coverage in Singapore**

Kelvin Bryan Tan^1^, Woan Shin Tan^2^, Marcel Bilger^3^ and Calvin WL Ho^4^

^1^Policy Research and Economics Office, Ministry of Health, Singapore

^2^Health Services & Outcomes Research, National Healthcare Group, Singapore

^3^Health Services & Systems Research Program, Duke-National University of Singapore, Singapore

^4^Centre for Biomedical Ethics, Yong Loo Lin School of Medicine, National University of Singapore, Singapore

*Corresponding author: Kelvin Bryan Tan

Email: kelvin_bryan_tan@moh.gov.sg

**This paper is the full country case study to accompany the summary paper “Monitoring and Evaluating Progress towards Universal Health Coverage in Singapore” that is part of the Universal Health Coverage Collection. Not commissioned; externally reviewed.**

**Abstract:** Promoting universal health coverage (UHC) has been an important part of Singapore’s overall development strategy, with a strong policy focus on the promotion, prevention and treatment of non-communicable diseases. The health care system is buttressed by a financing structure comprising the national medical savings scheme (Medisave), health insurance scheme (MediShield), and government subsidies to protect against unpredictable high expenditures.

While Singapore does not have a UHC monitoring framework per se, indicators on accessibility, quality and affordability of healthcare are tracked. They include tracer indicators for health-related Millennium Development Goals, and chronic conditions and injuries (CCI) recommended by the WHO and World Bank. Measures of disease burden, effective coverage, and affordability are also reported. Data sources include the National Health Survey (NHS), Household Expenditure Survey, and government and health facility information systems.

Life expectancy improved and premature mortality due to cancer, ischemic heart disease and stroke reduced over the years. Treatment coverage for chronic diseases (diabetes: 97%, hypertension: 97%, high blood cholesterol: 87%) was high and disease control was maintained for two-thirds of the cases. Based on NHS 2010, service coverage and treatment outcomes for diabetes and high blood cholesterol fared better for lower educated persons. For hypertension, service coverage was higher for the lower-educated but outcomes were worse. For cancer, knowledge and utilisation of screening increases with education attainment. The affordability indicators showed that MediShield coverage increased from 80% to 92% between 2007 and 2012. Household out-of-pocket health care payments made up 4.3-4.5% of income across all quintiles.

The choice of appropriate indicators will have to evolve as countries go through different phases of socioeconomic development and epidemiological change. As Singapore progresses, our challenge lies in ensuring that care remains accessible and affordable, and health outcomes are maintained and remains equitable across socioeconomic groups with population ageing.

**Summary Points:**

1. Promoting UHC has been an important part of Singapore’s overall development strategy. Currently, there is a strong policy focus on the promotion, prevention and treatment of non-communicable diseases (NCDs), and ensuring access to affordable health services.
2. While there is no UHC monitoring framework per se, Singapore’s philosophy of promoting universal access to and affordability of care has ensured that key indicators are embedded in the assessment of the health system. Indicators on accessibility, quality and affordability of healthcare for Singaporeans are regularly tracked and reported to Parliament as part of the Key Performance Indicators for the Ministry of Health. Equity-related considerations are incorporated into these indicators.
3. In the last two decades, life expectancy and health-adjusted life expectancy have improved. Cross-sectionally, service coverage and outcomes for individuals with diabetes and high blood cholesterol are better for the lower educated. For hypertension, service coverage was better for the lower-educated but outcomes performed worse. For asthma, there was an uneven SES gradient. For cancer, knowledge and utilisation of screening increases with education attainment.
4. The key challenge that Singapore faces going ahead is ensuring that good health outcomes continue to be achieved with population aging and projected increases in chronic conditions. The close monitoring of SES gradients in risk factors and medical treatment makes certain that these copayments do not discourage use of important primary care and prevention that contribute in lowering the risk factors for these costly chronic conditions.
5. The example of Singapore illustrates that even for a country with an extensive health care system, monitoring of service coverage and financial protection still remains highly important. The choice of appropriate indicators will have to evolve as countries go through different phases of socioeconomic development and epidemiological change. To provide effective feedback on the performance of the health system, the indicators should be tailored to each country’s unique situation, as well as ethical and policy goals.
6. Nevertheless, for the purpose of benchmarking and sharing across countries, it is also important to have a common set of indicators. Given that equity in both coverage and financial protection is important, the lack of a globally agreed upon definition of equity and population strata needs to be addressed.

**1. Background**

The Republic of Singapore is an island-state in Southeast Asia. The country is 716 square kilometres and is highly urbanized with a total population of 5.4 million in 2013. The population is multiracial and made up of individuals of Chinese (74%), Malay (13%) and Indian (9%) ethnicity. Since achieving independence in 1965, Singapore has managed to put in place a healthcare infrastructure and financing framework that allowed the attainment of one of the world’s best health outcomes, with a healthcare system that is affordable to current users and sustainable for future generations.

Inheriting a British NHS-like healthcare system, health care in Singapore has been provided by public hospitals and outpatient clinics since independence in the 1960s. A rapid expansion of public sector health care services and infrastructure took place in the 1960s and 1970s [1],[2]. Table S1 highlights the milestones in the development of the Singapore healthcare system. With rapid economic growth, resources were invested into providing better housing, clean water, improved sanitation, and good education combined with better nutrition to improve the health status of its population [4]. Early investments in health promotion, prevention, and public education played an important role in raising the life expectancies of Singaporeans. Infant mortality rate dropped from 35.6 per 1,000 live births in 1960 to 2 per 1,000 in 2011. During this period, average life expectancy at birth increased from 66 years old to 82 years old (Figure S1).


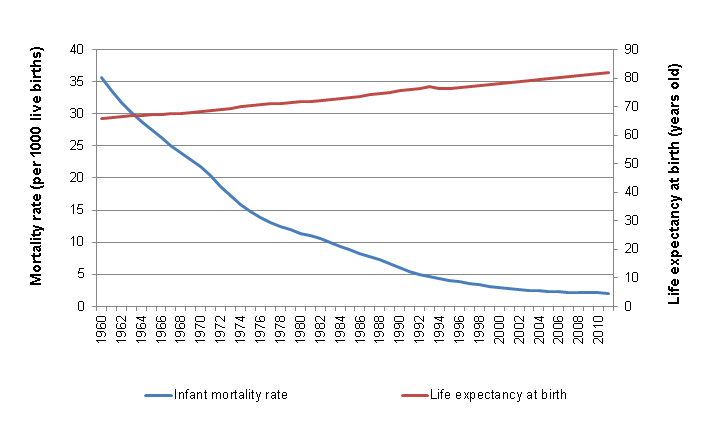


**Figure S1**: Life Expectancy and Infant Mortality, Singapore, 1960 – 2011.

Data Source: Department of Statistics

| **Period** | **Milestones** |
| --- | --- |
| 1960s | Building of new public general hospitals |
|  | Rapid expansion of obstetrics and gynaecological services |
|  | Island-wide inoculation programme |
|  | School Dental Clinic initiative was set up |
|  | Public education programmes on effects of littering through campaigns |
| 1970s | Nutrition programme started in the Maternal and Child Health clinics |
|  | Introduction of one-stop primary care clinics (polyclinics) |
|  | Committee for Postgraduate Medical Education was set up in 1970 to increase number of specialists |
| 1980s | A special clinic for obese school children was set up |
|  | Expansion of tertiary services |
|  | Encourage self-reliance with implementation of Medisave in 1984 |
|  | Started to restructure the public hospital system, granting more autonomy to hospitals and promoting innovation |
| 1990s | Medishield was implemented in 1990 to provide financial coverage for catastrophic illnesses, |
|  | Medifund was implemented in 1993 as a safety net for those who cannot afford the subsidised bill charges  Liberalisation of use of Medisave to pay for premiums of Private Medical Insurance Schemes (PMIS) in 1994 helps to further open up the private market for health insurance. |
|  | Promotion of good nutrition in recognition of chronic degenerative diseases such as heart disease, stroke and cancer |
| 2000s onwards | ElderShield, an insurance scheme, was introduced in 2002 to help elderly persons who become severely disabled  Eldercare fund was set up in 2000 to fund means-tested subsidies to the intermediate and long-term care sector |
|  | Means-testing was implemented in 2009 for subsidised ward classes in public hospitals to focus subsidies to the lower-income groups |
|  | Liberalization of Medisave to meet the challenges of an ageing population - outpatient treatment of chronic diseases; community hospitals; hospices ; day rehabilitation centres |
|  | Expansion of the MediShield coverage of bills in 2005, 2008 and 2013. Reform in 2005 to restructure MediShield as the basic tier of the Private Medical Insurance Scheme (PMIS) plans. |
|  | Portable subsidies introduced in 2000 for privately provided primary care services for lower-income individuals |

**Table S1**: Milestones in the Development of the Singapore Healthcare System

The World Health Organisation defines Universal Health Coverage (UHC) as a situation in which all people who need health services receive them, without incurring financial hardship. Promoting universal health coverage (UHC) has been an important part of Singapore’s overall development strategy. Timely access to health services is crucial in promoting and sustaining health. As a country in which the health transition is well advanced, there is a strong policy focus on the promotion, prevention and treatment of non-communicable diseases (NCDs). At the same time, to ensure that individuals are able to promote access to affordable health services without incurring excessive financial hardship, the health care delivery system is buttressed by a financing system comprising medical savings, health insurance and government subsidies to offer protection against unpredictable high expenditures.

In this review, we will provide a background on the expansion of population and service coverage in Singapore and how the financing system has evolved to support changes in policy goals. Next, we will describe the monitoring and evaluation framework for NCD prevention and management as well as indicators used to measure the extent of financial protection. Specific indicators stratified by socioeconomic status will be presented and we will further discuss the results within the UHC context. Lastly, we will highlight the current gaps in the UHC monitoring and evaluation framework as well as how to establish a feedback loop to ensure that policies are process- and outcomes-oriented in relation to UHC attainment.

**2. Universal health coverage: the policy context**

**Non-Communicable Disease Burden**

With rapid population ageing, as well as the growing NCD burden, developed countries with an extensive health care system will struggle to finance healthcare expenditures going forward. Singapore is no exception. The number of Singapore residents aged 65 years or older is also projected to more than double from 404,400 (11%) in 2013 to 900,000 (19%) in 2030 [5],[6]. As one of the fastest ageing countries in Asia and the adoption of unhealthy lifestyles, NCDs such as cancer, ischemic heart disease and pneumonia together accounted for approximately 60% of the total causes of death in 2009. The four common chronic conditions affect about 1 million Singaporeans: diabetes mellitus, hypertension, hyperlipidaemia, and stroke [7].

Improving the management of individuals with chronic illnesses is an increasingly important item on the health policy agenda. Evidence-based primary as well as secondary prevention in the early stages of the chronic diseases before complications set in, have been shown to improve health outcomes and concurrently reduce the need for specialist attention and hospitalisation.

**Health Care Financing System in Singapore**

The *raison d'être* of the Singapore health care financing system may be broadly described as a shared responsibility among individuals and families, insurers and the government. Individuals and families should have a role in living healthily and in saving for future healthcare expenditures while health care providers are incentivized to deliver cost-effective care. In addition, insurers need to mitigate the financial risk associated with illness, and the government is responsible to provide a safety net by helping the poorest, sickest and most deserving.

Currently, Singapore spends approximately 4% of the Gross Domestic Product (GDP) on health care (Figure S2). The country has evolved a mixed financing system, with multiple tiers of protection in efforts to ensure that no Singaporean is denied access to basic healthcare because of affordability issues. Health care is provided by tax-financed government subsidies available to all Singapore citizens at public hospitals and public polyclinics. The application of income-based subsidies with differential co-payments for acute hospitalization, and intermediate and long-term care services reflect a philosophy of providing targeted assistance with higher income individuals paying higher user charges.

**
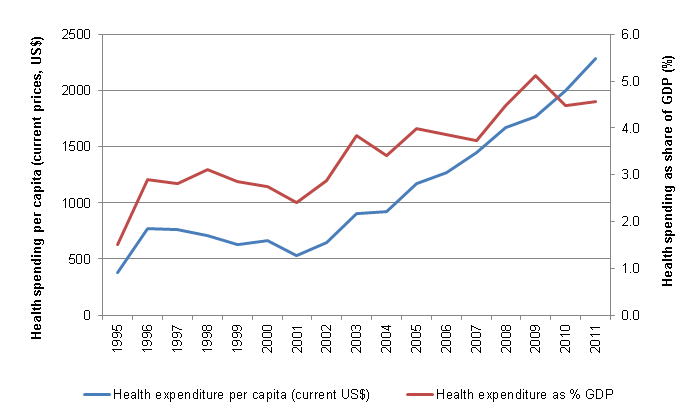
**

**Figure S2**: Health Spending as a percentage of GDP and per capita (current prices, US$), 1995 – 2011.

Data source: World Health Organization National Health Account database

However, to avoid the drawback of ‘free’ medical services stimulating insatiable demand and to ensure longer-term financial sustainability of the healthcare system, co-payments are a key feature of the Singapore healthcare financing system. Co-payments were introduced starting in 1960, with a co-payment of US$0.39 (using 2013 exchange rate of US$1 = S$0.79) for outpatient consultations but with the implicit understanding was that government would step in for those unable to afford healthcare. Value-based co-payments are used to direct access to effective medical services and limit access to services whose costs exceed the expected clinical gain [3]. While the concepts of value-based insurance design have only been recently proposed in the US, value-based differential co-payments were already implemented in Singapore from the 1960s. Vaccinations, school-health services were offered free or with very low co-payments. Public polyclinics were expanded to offer heavily subsidised services to all Singaporeans.

Rising wages and growing affluence allowed Singaporeans to afford better medical treatment and services. To enable people to save up to pay for such treatment, mandatory medical savings accounts (MSAs) were set up in 1984. As a prepayment scheme, Medisave allows individuals to spread their contributions to healthcare financing over a lifetime instead of having to pay for most of them post-retirement. MSAs alone do not suffice in the event of catastrophic medical expenses, and need to be complemented by high-deductible insurance plans. A government-run insurance scheme MediShield was set up to provide basic coverage, with private insurers allowed to provide coverage for inpatient stays with better physical amenities under private integrated shield plans. Outpatient treatments, such as kidney dialysis, and approved cancer treatments, such as chemotherapy and radiotherapy are also covered. In 2012, 92 % of the 3.29 million Singapore citizens were covered by MediShield and private integrated shield plans (Box S1). For individuals who were unable to accumulate sufficient Medisave because of their low income, means-tested scheme Medifund was introduced in 1998 to assist low-income families unable to pay the 20% co-payment in subsidised inpatients wards.

Therefore, UHC in Singapore can be understood as the financial protection provided by the government through direct subsidies as well as the 3Ms -- Medisave, MediShield and Medifund. Public healthcare institutions as a rule treat all patients regardless of their ability to pay. The 3Ms are thus meant to complement government subsidies to ensure that they are better targeted and fiscally sustainable in the long-run.

| **Box S1: Healthcare Financing Schemes in Singapore**   1. **Medisave**: The scheme was introduced in 1984 as a compulsory national savings scheme to help individuals set aside 6 to 8 % of their income to future personal or immediate family’s hospitalisation, day surgery and certain outpatient expenses. To discourage over consumption, Medisave withdrawals are subjected to a daily limit for hospital charges and a fixed limit for surgical operations. Medisave places responsibility on the individual and the family to save and utilize funds carefully by emphasising the use of savings to finance the consumption of healthcare services. 2. **MediShield**: Since Medisave does not adequately provide for catastrophic or chronic illnesses, Medishield, a low-cost catastrophic illness insurance scheme, was introduced in 1990. All Singaporeans aged 90 and below are eligible to buy Medishield insurance, with premiums payable through their Medisave accounts. In order to circumvent the moral hazard problem, there is a maximum amount that a member can claim for as well as an annual deductible that members have to pay out using cash or their Medisave savings. Integrated Shield Plans were introduced in July 2005 to supplement basic MediShield coverage for individuals who opt for additional benefits and coverage. In November 2013, the Ministry of Health further announced that a committee has been set up to review the MediShield scheme to ensure universal coverage regardless of age and pre-existing illnesses [8]. 3. **Medifund**: In April 1993, Medifund was established to help needy Singaporeans pay their medical bills. It provides a safety net for those, who, despite help from government subsidies and Medisave and Medishield, are still unable to afford their medical expenses. Note that only the interests generated by the approximately S$3 billion fund (FY 2012), not the capital, may be disbursed. Eligibility for Medifund assistance is subjected to means-testing administered by medical social workers for inpatient and outpatient services at all public hospitals and community hospitals, nursing homes and hospices. In the financial year 2011, $84.3 million were disbursed for a total of 518,389 applications, among which, 93 % received full assistance for the outstanding subsidised bill [9]. |
| --- |

**Healthcare Facilities**

In 2012, there were a total of about 10,756 hospital beds in the 25 hospitals and specialty centres. About 85% of the beds are in the 15 public hospitals and specialty centres. The 8 public hospitals comprise 6 acute general hospitals, a women's and children's hospital and a psychiatry hospital. The general hospitals provide multi-disciplinary acute inpatient and specialist outpatient services and a 24-hour emergency department. In addition, there are 6 national specialty centres for cancer, cardiac, eye, skin, neuroscience, dental care and a medical centre for multiple disciplines. The 18 public primary care clinics provide approximately 20% of primary care services consumed with 2,400 private medical practitioner's clinics providing the remaining 80%. Patients are free to choose and move freely between primary care providers.

General consultation fees at the government primary care clinics are US$0.87-0.89 and US$4.8-5.1 for adults and child/elderly respectively. (using 2013 exchange rate of US$1 = S$0.79). Patients referred by government primary care clinics to public hospital specialists are subsidised at 50 %. Within the public acute hospital sector, patients may choose to be admitted to single-bedded unsubsidised (Class A), two-bedded partially subsidised (Class B1) or dormitory-styled heavily subsidised (Class B2 and C) wards. Class B2 and C wards are fan-ventilated 6-bedded and 8-bedded wards respectively with shared bathroom facilities and amenities. Within the income-based subsidy framework, individuals with incomes falling below a predetermined threshold would pay only 20% of the bill if they had chosen Class C wards. For most individuals, this co-payment is fully covered with Medisave and MediShield. For individuals who had chosen unsubsidised wards, they would pay the full costs with a combination of Medisave, cash and private insurance. These income-differentiated copayments ensure that government subsidies are targeted at the needy and sick.

**3. Monitoring and evaluation for UHC**

While there is no UHC monitoring framework per se, indicators on accessibility, quality and affordability of healthcare for Singaporeans are regularly tracked and reported to Parliament as part of the Key Performance Indicators (KPI) for MOH [10]. In tracking health sector progress and performance in meeting healthcare priorities as set out in the Healthcare 2020 Masterplan [11], they also serve to monitor the sustainability and efficacy of UHC provision as well as to identify areas of intervention which could improve the population health of Singaporeans.

The list of indicators (Table S2) include many of the potential tracer indicators for both health-related MDGs (e.g. vaccination coverage for Diphtheria and Measles) and chronic conditions and injuries (CCI) (premature mortality from cancer, ischaemic heart disease and stroke and prevalence of obesity, diabetes, psychiatric morbidity) as recommended by WHO and World Bank [12]. These tracer indicators have been selected because diabetes mellitus, ischemic heart disease and stroke were the top three leading causes of premature death and ill-health in Singapore, accounting for 28 % of total disability-adjusted life years lost [13].

| **Indicator/Year** | **2007** | **2008** | **2009** | **2010** | **2011** | **2012** |
| --- | --- | --- | --- | --- | --- | --- |
| **Health of Singaporeans** | | | | | | |
| Life Expectancy, Female | 83.2 | 83.3 | 83.7 | 84.1 | 84.3 | 84.7 |
| Life Expectancy, Male | 78.4 | 78.4 | 79.0 | 79.3 | 79.6 | 80.0 |
| Infant Mortality | 2.1 | 2.1 | 2.2 | 2.0 | 2.0 | 2.0 |
| Age-standardized premature mortality rate from cancer per 100,000 residents aged 35-64 | 107.2 | 112.2 | 104.0 | 102.6 | 102.7 | 105.0 |
| Age-standardized premature mortality rate from ischaemic heart disease per 100,000 residents aged 35-64 | 55.2 | 59.2 | 51.6 | 49.9 | 45.0 | 44.1 |
| Age-standardized premature mortality rate from stroke per 100,000 residents aged 35-64 | 17.6 | 17.0 | 14.2 | 17.0 | 16.4 | 16.0 |
| % of Singaporeans aged 18-69 who smoke (National Smoking Control Programme) * | 13.6 | NA | NA | 14.3 | NA | NA |
| Prevalence of obesity (Body Mass Index ≥ 30 kg/m2) among Singaporeans (aged 18-69) (%) * | 6.9 | NA | NA | 10.8 | NA | NA |
| Prevalence of diabetes in Singaporeans (aged 18-69) (%) | 8.2 | NA | NA | 11.3 | NA | NA |
| Prevalence of high total cholesterol (≥ 6.2 mmol/L) among Singaporeans (aged 18-69) (%) * | 18.7 | NA | NA | 17.4 | NA | NA |
| Percentage of Singaporeans aged 20-59 years with minor Psychiatric Morbidity (e.g. anxiety, depression and related disorders) * | 9.5 | NA | NA | NA | NA | 13.4 |
| % of children aged 2 years who have undergone vaccination for a) Diphtheria - vaccinated with the 1st, 2nd and 3rd dose of the Diphtheria vaccine; | NA | 96.9 | 96.8 | 96.1 | 96.0 | 95.0 |
| % of children aged 2 years who have undergone vaccination for b) Measles - vaccinated with the 1st dose of the measles vaccine | NA | 94.9 | 95.2 | 95.1 | 95.2 | 95.0 |
| **Access to Care**** | | | | | | |
| Patients who waited ≤ 40 minutes for registration at Polyclinics (%) | 95.7 | 94.8 | 95.3 | 95.2 | 91.0 | 88.9 |
| Patients who waited ≤ 100 minutes for consultation at Polyclinics (%) | 95 | 94.3 | 94.4 | 92.8 | 92.7 | 93.6 |
| % of Patients who waited ≤ 60 days for new subsidised Specialist Outpatient Clinics appointment | NA | 79.8 | 80.7 | 83.1 | 84.9 | 86.3 |
| Acute hospital 30-day readmission rates | 11.2 | 11.0 | 11.7 | 11.5 | 11.3 | 11.9 |
| **Affordability of Care** | | | | | | |
| Proportion of Singaporeans covered by MediShield and Integrated Shield Plans (%) | 80 | 84 | 88 | 90 | 92 | 92 |
| Average Coverage by Medisave & MediShield bills for Class B2/C wards (%) |  |  | 95 | 94 | 92 | 92 |

* National Health Survey, Singapore, various years

** As a share of all patients attending the Polyclinics and Specialist Outpatient Clinics

**Table S2:** Singapore Health System Key Performance Indicators

Data Source: Ministry of Finance, Singapore Budget 2012

WHO and World Bank’s recommendation on ensuring service coverage for the bottom 40% income distribution is also implicit in the accessibility and affordability indicators reported to parliament. Lower income patients are seen at public polyclinics and subsidised specialist outpatient clinics and hence measures of waiting time at these facilities provide an indication of access to basic medical services – all patients are guaranteed same day visits to primary care doctors and to specialists within a reasonable time frame of two months.

The affordability indicators also emphasize Medisave and MediShield coverage of inpatient bills at Class B2/C wards which attract lower income patients because they are highly subsidised. Coverage of Singaporeans with MediShield and Integrated Plans are also tracked as an indicator of how sustainable the healthcare financing system is. The greater such coverage, the less the state will have to step in to assist the poor.

These indicators are measured from a combination of population-based health and household expenditure surveys, national medical claims database and health facility-based administrative databases. Some of these data sources (Box S2) allow for further breakdown by age, gender and socioeconomic status (geographical differences are less important in Singapore due to the small size of the country). For the purpose of targeted interventions, morbidity and mortality changes over time can be broken down by broad disease groups [14].

Stratification by socioeconomic status (SES) poses particular difficulties especially in a country that has seen rapid development. As older cohorts tend to be less educated and have lower income, a negative SES-Health gradient may be simply due to the fact that the elderly are less healthy, rather than that those with lower SES have less access to healthcare.

In addition, these countries are often characterized by intergenerational transfers as older generations invest in their children’s education, with the expectation of receiving financial support in their old age. Such social norms are reflected and supported by government policy in assessing health care affordability by accounting for the income of immediate family members in addition to that of other household members. In government surveys in Singapore, a household is defined as a group of two or more persons living together in the same house and sharing common food or other arrangements for essential living. Therefore, only income and expenditures of those staying in the same household are captured, and therefore might understate the affordability of healthcare for families who are able to draw on the financial resources of family members who are not residing with them.

Lastly, one of the key limitations is that clinical and epidemiological data is often not linked to financial data at the patient level. This makes it difficult to infer that low service coverage is due to financial unaffordability, or to design interventions to improve coverage and health outcomes.

| **Box S2: Different Sources of Data for Measuring Progress towards UHC**  System-level performance of Singapore’s healthcare sector can be measured using the healthy life expectancy (HALE) or disability-adjusted life-years (DALY) published in the Global Burden of Disease study 2010 [14]. A cross country comparison of these indicators allows policymakers to benchmark health outcomes at a macro-level internationally. Longitudinal monitoring of these indicators and relative international ranking specify the absolute and relative improvement (deterioration) of our health system performance.  To conduct within-country monitoring and evaluation of the extent of intervention coverage and financial protection, three main data sources are used:   1. National Health Survey (NHS): The 6-yearly survey is conducted by the Ministry of Health (MOH) and collects information on the prevalence of major NCDs and related risk factors like obesity and smoking. It also includes information on the coverage of chronic disease screening, use of primary healthcare services, mental health and self-rated overall health. Till date, surveys have been completed for 1992, 1998, 2004 and 2010. The survey findings are used by MOH to monitor the health of the population, track progress towards national health targets and for planning of services [15].   One key advantage of the NHS is the use of health screening including biochemistry laboratory tests to ascertain disease prevalence. The NHS is able to ascertain disease prevalence and thereby deduce population need with higher accuracy, which is important for the derivation of effective intervention coverage indicators.   1. Household Expenditure Survey (HES): The first HES was undertaken in 1956/57 and covered only the urban area. The second survey covering the whole country was carried out in 1972/73. This 5-yearly survey is conducted by the Department of Statistics and collects information from resident households in Singapore on their expenditure, income, savings and selected household characteristics [16]. Households selected for the survey are provided with an expenditure diary to record daily household and personal expenditure over a period of two weeks. Data from MOH’s health information system were however used to derive hospitalization expenditures. Comprehensively, health expenditures include spending on medical products, appliances and equipment; outpatient services; and hospital, convalescent and rehabilitation services. One weakness of the survey is that it did not require respondents to distinguish between expenditures paid for using Medisave, cash or subsequently reimbursed by third party payers. Thus, the results do not accurately measure the out-of-pocket (OOP) burden. 2. Government and Health Facility Information Systems: As the Medisave and the Medishield schemes are administered by the Central Provident Fund Board (CPFB), population coverage indicators are tracked using data from CPFB’s information system. Claims data and hospitalization data obtained from individual acute public and private hospitals are used by MOH to monitor the extent of financial coverage offered by these schemes. |
| --- |

**4. Progress towards UHC in Singapore**

Top-line indicators for Singapore’s performance show a steady improvement in life expectancy and reduction in premature mortality due to cancer, ischemic heart disease and stroke (Table S2). Reflecting this overall decrease in NCD burden, HALE at birth improved from 1990 to 2010 for both males and females. Comparatively, in the same timeframe, Singapore’s international ranking rose from eleventh to second for males, and from fourteen to four for females (Table S3).

|  | 1990 | Male | 2010 | Male |  | 1990 | Female | 2010 | Female |
| --- | --- | --- | --- | --- | --- | --- | --- | --- | --- |
| Rank | Country | HALE | Country | HALE | Rank | Country | HALE | Country | HALE |
| 1 | Japan | 66.6 | Japan | 68.8 | 1 | Japan | 70.0 | Japan | 71.7 |
| 2 | Andorra | 65.4 | Singapore | 68.1 | 2 | Spain | 68.2 | South Korea | 70.3 |
| 3 | Cyprus | 64.6 | Switzerland | 67.5 | 3 | Andorra | 68.2 | Spain | 70.1 |
| 4 | Kuwait | 64.5 | Spain | 67.3 | 4 | Switzerland | 68.0 | Singapore | 70.0 |
| 5 | Malta | 64.2 | Italy | 66.8 | 5 | France | 67.4 | Taiwan | 69.6 |
| 6 | Greece | 64.2 | Australia | 66.8 | 6 | Italy | 67.3 | Switzerland | 69.5 |
| 7 | Switzerland | 64.1 | Canada | 66.7 | 7 | Canada | 67.2 | Andorra | 69.3 |
| 8 | Israel | 64.1 | Andorra | 66.7 | 8 | Australia | 67.0 | Italy | 69.1 |
| 9 | Sweden | 64.1 | Israel | 66.7 | 9 | Sweden | 67.0 | Australia | 69.0 |
| 10 | Spain | 64.0 | South Korea | 66.7 | 10 | Cyprus | 66.8 | France | 68.8 |
| 11 | Singapore | 64.0 |  |  | 14 | Singapore | 66.6 |  |  |

**Table S3**: Healthy Life Expectancy by Sex, 1990 and 2010

Data source: Salomon JA *et al.* Healthy life expectancy for 187 countries, 1990-2010: a systematic analysis for the Global Burden Disease Study 2010. Lancet. 2012 Dec 15;380(9859):2144-62.

What is of some concern is the increasing prevalence of smoking, obesity and diabetes from 2007 to 2010. Breakdown from the most recent 2010 survey indicate that these prevalence rates follow a negative SES gradient (Table S4). A negative SES gradient was also observed in relation to the prevalence of diabetes, hypertension, high cholesterol, cataract, arthritis, and hearing loss. The lower educated groups also reported lower rates of leisure-time exercise. The positive association between education and physical activity is consistent with previous Western studies [17]-[19]. However, higher educated individuals reported higher rates of mental health issues and asthma. Other studies have similarly found positive association between socioeconomic status and prevalence rates of asthma [20].

| **Prevalence rate** | **Primary & below** | **Secondary** | **Post-Secondary** | **Total** |
| --- | --- | --- | --- | --- |
| **Health Risk Factors** | | | | |
| Obesity, BMI: ≥ 30.0 | 11.6% | 12.1% | 9.7% | 10.8% |
| Cigarette smoking * | 21.4% | 18.3% | 9.6% | 14.3% |
| Alcohol consumption ** | 4.8% | 2.7% | 1.8% | 2.6% |
| Leisure-time regular exercise + | 15.1% | 16.5% | 21.7% | 19.0% |
| **Non-Communicable Diseases** | | | | |
| Diabete mellitus | 19.1% | 14.9% | 6.5% | 11.3% |
| Hypertension ^ | 37.7% | 23.3% | 16.9% | 23.5% |
| High total cholesterol | 45.2% | 29.7% | 19.2% | 26.9% |
| Current asthma | 2.6% | 4.5% | 4.0% | 3.9% |
| Poor mental health | 8.3% | 11.9% | 15.0% | 12.9% |
| Diagnosed cataract | 10.1% | 5.7% | 2.5% | 4.8% |
| Diagnosed arthritis | 6.6% | 3.1% | 1.9% | 3.1% |
| Hearing loss | 10.4% | 10.7% | 8.1% | 9.3% |

BMI: Body Mass Index

Denominator: 18-69 years old resident adults

* Cigarette smoking: smoked cigarettes at least once a day

** Alcohol consumption: > 4 times per week

+ Leisure-time regular exercise: exercised ≥ 20 mins for ≥ 3 days per week

^ Hypertension: Denominator comprise 30-69 years old individuals

**Table S4:** Age-Standardized Prevalence of Health Risk Factors and Non-Communicable Diseases.

Data source: National Health Survey 2010

While UHC may not equalize the prevalence of diseases across SES due to the influence of various determinants of health, it should ensure that intervention coverage are on par across SES. NHS results reveal heterogeneous SES gradients: When it comes to diabetes and high cholesterol, those with primary and below education individuals had higher service coverage (in terms of those with more than one doctor visit) and better outcomes than post-secondary individuals. Service coverage for hypertension was also better for the lower-educated, even though outcomes were less favourable. For asthma, there is an uneven SES gradient, with post-secondary individuals achieving best coverage and outcomes followed by primary and below and then secondary educated individuals. For cancer, knowledge and utilization of screening increases with education attainment (Table S5). Other studies have reported that women in low social classes tend to have lower screening participation rates than those in higher classes. [21]

|  | **Primary and below** | **Secondary** | **Post-Secondary** | **Total** |
| --- | --- | --- | --- | --- |
| **Diabetes Mellitus** | | | | |
| ≥ 1 doctor visit in past 1-year for diabetes in known population | 99.3% | 99.4% | 90.5% | 97.4% |
| % acceptable blood sugar control in known population | 74.5% | 61.8% | 68.6% | 68.0% |
| **Hypertension** | | | | |
| ≥ 1 doctor visit in past 1-year for hypertension in known population | 98.0% | 96.6% | 97.2% | 97.3% |
| % acceptable blood pressure control in known population | 62.5% | 69.0% | 73.6% | 68.0% |
| **High Blood Cholesterol** | | | | |
| ≥ 1 doctor visit in past 1-year for hyperlipidemia in known population | 93.8% | 83.5% | 83.7% | 86.8% |
| % acceptable total cholesterol control in known population | 67.4% | 68.5% | 54.0% | 63.1% |
| **Asthma** | | | | |
| % with no asthma episode in past 1-year among those with current asthma | 45.3% | 36.2% | 56.8% | 47.9% |
| % long-term preventive medication daily among those with current asthma | 49.2% | 34.3% | 66.8% | 50.0% |
| **Breast Cancer Screening** | | | | |
| Knowledge of mammography | 87.2% | 91.6% | 98.0% | 90.9% |
| Ever undergone mammography | 61.8% | 66.2% | 77.0% | 66.3% |
| Had mammography within past 2-years | 29.7% | 41.0% | 59.8% | 39.6% |
| **Cervical Cancer Screening** | | | | |
| Knowledge of pap smear | 69.3% | 92.8% | 91.6% | 87.1% |
| Ever undergone pap smear | 67.4% | 75.9% | 69.7% | 71.3% |
| Had pap smear within past 3-years | 34.3% | 51.6% | 52.0% | 47.9% |
| **Colorectal Cancer Screening** | | | | |
| Ever had faecal occult blood test (FOBT) | 20.1% | 24.5% | 44.6% | 27.8% |
| Had FOBT within past 1-year | 4.8% | 10.2% | 19.1% | 10.3% |

**Table S5:** Service and Effective Intervention Coverage for Chronic Diseases by Educational Level.

Data source: National Health Survey 2010

Effective coverage is also captured by waiting times at public polyclinics and public hospitals (Accessibility indicators in Table S2). These indicators also help inform whether service provision needs to be expanded to meet demand. Waiting times for government subsidised primary care are within targets (≤ 40 minutes for registration, ≤100 minutes for consultation), and waiting times for subsidised specialist appointments (≤ 60 days for first appointment) have also improved overtime.

There is no explicit tracking of catastrophic payments or out-of-pocket spending vis-à-vis household resources in part because the health financing system has been structured to ensure that nobody is denied care because of affordability. The availability of subsidised health care in public hospitals and polyclinics are aimed at reducing the out-of-pocket spending. Patients with financial difficulty even after using their Medisave money and MediShield coverage can apply for Medifund. Eligibility to Medifund is based on the assessment of medical social workers. Those who do not qualify would have been assessed to have sufficient means within their immediate family and household to pay for their healthcare expenditures.

The efforts to increase MediShield coverage can be seen as one way to increase financial protection, and reduce the likelihood of low-income patients not seeking care. The affordability indicators show an increase in MediShield and Integrated plan coverage from 80 to 92 % (see affordability indicators in Table S2). The recent announcement to make MediShield mandatory will raise coverage to 100% when the scheme is implemented.

The declining coverage of Class B2/C bills by Medisave and MediShield is due to the fact that claim limits have to be continually raised in tandem with medical price inflation. They were last raised in 2008 and 2009. All Singaporeans are eligible for inpatient subsidies of up to 80% in Class C ward and 65% in Class B2 ward. Thus, out-of-pocket co-payment by the patients only comprise 1.6 % and 2.8% of the total charges given that Medisave and MediShield covered 92% of the post-subsidy bill. This is significantly lower than the upper-bound of 61% out-of-pocket share for National Health Expenditure [21].

While these indicators already focus on the bottom 40% of the population, it is useful to look at the relative burden of healthcare financing across various income quintiles. We had previously highlighted several limitations of household expenditure survey (i.e. not taking into account inter-generational transfers, not measuring family affordability and not capturing modes of payment). Nevertheless, the health-expenditures as a share of total household expenditure is a useful proxy indicator to measure financial burden of health expenditures on households [22]. The data in Table S6 suggests that although there is an increasing trend in the share of total expenditures spent on health-related items between 1997 and 2007, the rate of increase for the lowest quintile is slower than that for the higher income groups.

| **Resident Household Income Quintiles** | | | | | | |
| --- | --- | --- | --- | --- | --- | --- |
|  | **1st - 20th** | **21st - 40th** | **41st - 60th** | **61st - 80th** | **81st - 100th** | **Total** |
| 1997/98 | 5.0 | 3.0 | 3.4 | 3.2 | 3.3 | 3.5 |
| 2002/03 | 5.7 | 4.8 | 4.8 | 4.8 | 4.1 | 4.7 |
| 2007/08 | 6.0 | 5.5 | 5.5 | 5.3 | 4.8 | 5.3 |
| 2007/08 (OOP Estimate) | 4.3 | 4.3 | 4.3 | 4.5 | 4.3 | 4.3 |

*Averages are calculated excluding those with zero income

**Table S6:** Proportion of Monthly Household Expenditure Spent on Healthcare

Source: Household Expenditure Survey 1997/98, 2002/03, 2007/08. The OOP estimate for 2007/08 is derived from linking administrative data of Medisave and MediShield payouts with the Household Expenditure Survey.

One limitation in the interpretation of these results is that the expenditure here does not distinguish between what is paid for by cash vis-à-vis Medisave, MediShield insurance and Medifund. Taking these into account is likely to show a lower burden of financing for lower income families since Medisave and insurance coverage of their health expenditures are larger than for higher income families. To get a better estimate of the OOP cash component, we linked the Household Expenditure Survey with administrative data on Medisave and MediShield payouts. The final row of Table S6 reports the household healthcare expenditures excluding Medisave and MediShield payouts. Here we see that progressiveness of Medisave and MediShield, in reducing the OOP burden more for the lower income groups then the higher income groups to the extent that OOP as a proportion of Household Expenditure is flat across income groups. Note that the actual OOP cash payment is likely to be even lower since we still have not taken into effect of Medifund payments which are targeted at the lower income groups.

**5. Conclusions and recommendations**

The example of Singapore illustrates that even for a country with an extensive health care system, monitoring of service coverage and financial protection still remains highly important. Singapore’s experience also shows that the choice of appropriate indicators will have to evolve as countries go through different phases of socioeconomic development and epidemiological change. In such evolution, there remains room for improvement in setting appropriate indicators and putting in place the necessary means to track and monitor such indicators. To provide effective feedback on the performance of the health system, the indicators should be tailored to each country’s unique situation, as well as ethical and policy goals. Nevertheless, for the purpose of benchmarking and sharing across countries, it is also important to have a common set of indicators. Given that equity in both coverage and financial protection is important, the lack of a globally agreed upon definition of equity and population strata needs to be addressed.

Singapore’s philosophy of promoting universal access to and affordability of care has ensured that key indicators are embedded in the assessment of the health system. This includes monitoring population coverage and the impact of healthcare financing policies. As a country that has undergone tremendous epidemiological shifts in the last five decades, there is also a strong focus on tracking the effective coverage for NCDs that are highly prevalent in Singapore. In examining the KPIs reported to Parliament by the Ministry of Health every year, we find several of the tracer indicators on service coverage that have been recommended by WHO. The KPIs also include many “hardwired” equity considerations – access in terms of waiting times to subsidised primary care and specialist services, Medisave and MediShield coverage for subsidised inpatient services which are used by the lowest 40% of income distribution.

This survey has been compiled using data from surveys and information systems, which are not fully integrated with each other. Going ahead, there is a need to reconcile data definition and quality across different data sources. We will also need to examine the use of income as a SES stratifier. In a population that is rapidly ageing and characterized by retiree households that might still be well-supported financially by children who stay apart, earned income may not fully reflect purchasing power. There are some limitations to using education attainment as a proxy as well. Lastly, differentiating the mode of payment for healthcare expenditure in national surveys is also essential in allowing an assessment of the financial burden on individuals and households. Here a better tracking of patients with catastrophic payments would be helpful, not just for the purposes of UHC measurement, but also to inform efforts at improving existing coverage and financial safety nets.

The key challenge that Singapore faces going ahead is ensuring that good health outcomes continue to be achieved with population aging and projected increases in chronic conditions. To keep UHC fiscally sustainable, Singapore relies on a relatively innovative method of income and service-differentiated patient copayments. These copayments have to be continually adjusted to make certain that they do not discourage use of important primary care and prevention that contribute in lowering the risk factors for these costly chronic conditions. The close monitoring of SES gradients in risk factors and medical treatment that we report in this paper will thus be important. If Singapore is successful in ensuring that these gradients do not deteriorate, this aspect of Singapore’s health financing system could be instructive for other countries.

| **Box S3: Recommendations**   1. Develop globally agreed upon definitions of UHC service and financial coverage for the purposes of cross country comparisons. 2. Track the extent of families and individuals with catastrophic out-of-pocket health expenditure, especially for the elderly, sick and other vulnerable groups. . 3. Monitor Socioeconomic Status gradients in risk factors and effective coverage of health interventions to ensure that copayments do not deter treatment and other health-seeking behaviour. |
| --- |

**References**

1. Lim MK. (1998, March) Health care systems in transition. II. Singapore, Part I. An overview of health care systems in Singapore. J Public Health Med; 20(1):16-22.
2. Haseltine W. (2013) Affordable Excellence: The Singapore healthcare story. Singapore: Ridge Books.
3. Chernew ME, Rosen AB, Fendrick AM. (2007, March-April) Value-based insurance design. Health Aff (Millwood); 26(2):w195-203.
4. Abeysinghe, T., Himani, Lim, J. (2011) Equity in Singapore’s healthcare financing, Challenges for the Singapore Economy after the Global Financial Crisis, edited by P. Wilson, Singapore: World Scientific.
5. Department of Statistics, Singapore. (2013) Population Trends 2013. Republic of Singapore: Department of Statistics, Ministry of Trade and Industry. Retrieved from <http://www.singstat.gov.sg/publications/publications_and_papers/population_and_population_structure/population2013.pdf> Accessed November 18, 2013.
6. Committee on Ageing Issues. (2006, February 3) Report on the Ageing Population, Five-year Masterplan. Retrieved from <http://app.msf.gov.sg/portals/0/summary/research/cai_report.pdf> Accessed November 18, 2013.
7. Ministry of Health, Singapore. (2006, April 17) Better health outcomes for the chronically-ill through structured disease management programmes. Retrieved from <http://www.moh.gov.sg/content/moh_web/home/pressRoom/pressRoomItemRelease/2006/better_health_outcomes_for_the_chronically-Ill_through_structured_disease_management_programmes.html> Accessed November 18, 2013.
8. Ministry of Health, Singapore. (2013) Together, Better Healthcare for All: Medishield Life. Retrieved from <http://www.moh.gov.sg/content/moh_web/home/pressRoom/Current_Issues/2013/national-day-rally-2013/medishield.html> Accessed January 7, 2009.
9. Ministry of Health, Singapore. (2013) Medical Endowment Scheme: Annual Report 2011/2012. Republic of Singapore: Ministry of Health. Retrieved from <http://www.moh.gov.sg/content/dam/moh_web/PressRoom/Current_Issues/2013/23%20Feb%202013%20-%20Medifund%20FY11%20Annual%20Report.pdf> Accessed January 8, 2014.
10. Ministry of Finance, Singapore. (2012) Singapore Budget 2012. Republic of Singapore: Ministry of Finance. Retrieved from <http://www.mof.gov.sg/budget_2013/revenue_expenditure/attachment/35%20MOH%202013.pdf> Accessed November 18, 2013.
11. Ministry of Health, Singapore. (2012) Healthcare 2020: Improving accessibility, quality and affordability for all Singaporeans. Healthscope. Retrieved from <http://160.96.2.237/content/dam/moh_web/healthscope/In%20this%20issue/Healthscope-COS%20Rv1.pdf> Accessed November 18, 2013.
12. Boerma, T. (2013, October 3) Measurement and Monitoring of Universal Health Coverage . Geneva: World Health Organization. Retrieved from

<http://www.who.int/healthinfo/UHC_RSM_Presentation_Oct2013.pdf>

Accessed November 18, 2013.

1. Phua HP, Chua AV, Ma S, Heng D, Chew SK. (2009, May) Singapore's burden of disease and injury 2004. Singapore Med J. 50(5): 468-78.
2. Salomon JA, Wang H, Freeman MK, Vos T, Flaxman AD, Lopez AD, Murray CJ. (2012 Dec 15) Healthy life expectancy for 187 countries, 1990-2010: a systematic analysis for the Global Burden Disease Study 2010. Lancet. 380(9859):2144-62.
3. Ministry of Health, Singapore. (2011) National Health Survey 2010. Republic of Singapore: Epidemiology and Disease Control Division, Ministry of Health. Retrieved from <http://www.moh.gov.sg/content/dam/moh_web/Publications/Reports/2011/NHS2010%20-%20low%20res.pdf> Accessed November 18, 2013.
4. Department of Statistics, Singapore. (2009) Report of the Household Expenditure Survey 2007/2008. Republic of Singapore: Department of Statistics, Ministry of Trade and Industry. Retrieved from <http://www.singstat.gov.sg/Publications/publications_and_papers/household_income_and_expenditure/hes2007.pdf> Accessed November 18, 2013.
5. Bennett S. (1995) Cardiovascular risk factors in Australia: trends in socioeconomic inequalities. J Epidemiol Community Health. 49:363-72.
6. Luepker RV, Rosamond WD, Murphy R, et al. (1993) Socioeconomic status and coronary heart disease risk factor trends. The Minnesota Heart Survey. Circulation. 88:2172-9.
7. Garrison RJ, Gold RS, Wilson PW, Kannel WB. (1993) Educational attainment and coronary heart disease risk: the Framingham Offspring Study. Prev Med; 22:54-64.
8. Farfel A, Tirosh A, Derazne E, Garty BZ, Afek A. (2010) Association between socioeconomic status and the prevalence of asthma. Ann Allergy Asthma Immunol. Jun;104(6):490-5.
9. Segnan N. (1997) Socioeconomic status and cancer screening. IARC Sci Publ. 1. (138):369-76.
10. Ministry of Health, Singapore. (2013) Healthcare Financing Sources. Republic of Singapore: Ministry of Health.

<http://www.moh.gov.sg/content/moh_web/home/pressRoom/Parliamentary_QA/2013/healthcare-financing-sources0.html> Accessed November 18, 2013.
